# A Cross-Sectional Analysis of Intimacy Problems, Stress Levels, and Couple Satisfaction among Women with Thrombophilia Affected by Recurrent Pregnancy Loss

**DOI:** 10.3390/ijerph20021208

**Published:** 2023-01-10

**Authors:** Razvan Nitu, Radu Neamtu, Olivera Iordache, Lavinia Stelea, George Dahma, Geanina Sacarin, George Socol, Aris Boarta, Carmen Silaghi, Daiana Puichita, Mircea Mihai Diaconu

**Affiliations:** Department of Obstetrics and Gynecology, “Victor Babes” University of Medicine and Pharmacy Timisoara, 300041 Timisoara, Romania

**Keywords:** recurrent pregnancy loss, thrombophilia, stress levels, marital attitudes, sexual dysfunction

## Abstract

Recurrent pregnancy loss (RPL) is one of the most challenging and difficult areas of reproductive treatment due to the immense emotional suffering inflicted on families and couples affected by RPL. As a result, it is predicted that couples experiencing recurrent pregnancy loss would have an increase in marital problems, stress levels, and anxiety, preventing them from achieving their family goals. The current cross-sectional study aimed to target pregnant women with thrombophilia with a history of RPL to observe their intimacy problems, stress levels, and couple satisfaction by completing a series of digital questionnaires. These patients were considered as the reference group, while the control group was formed by other women with thrombophilia and a history of RPL who eventually achieved pregnancy and gave birth. A total of 238 complete questionnaires were recorded (157 in the reference group and 81 in the control group). It was observed that women in the reference group who did not give birth had a significantly higher proportion of three or more pregnancy attempts (54.1% vs. 39.5%) and a significantly higher proportion of three more pregnancy losses (68.8% vs. 55.6%). It was observed that patients in the reference group were more likely to be emotion-oriented (42.7% vs. 27.2%). Also, women in the reference group had higher levels of dissatisfaction and lower levels of self-acceptance, pleasure, and marital quality scores. The total SII and DSCS scores were significantly lower than women with thrombophilia with a history of RPL who eventually gave birth. Women from the reference group had significantly greater intimacy problems and stress levels while having lower openness scores and self-esteem scores than women in the control group. It is possible that women with thrombophilia and recurrent pregnancy loss are more dissatisfied with their marriages than those who subsequently had one child. Since the financial status of those who achieved pregnancy was observed to be higher, it is likely that they achieved pregnancy by ART interventions, as they reported in questionnaires. It is important to target families afflicted by thrombophilia and other reasons for infertility to ease their access to ART therapies. By achieving their objectives, affected families will minimize dissatisfaction, divorce rates, and stress.

## 1. Introduction

Recurrent pregnancy loss (RPL) is one of the most difficult and demanding areas of fertility medicine because of the enormous emotional load imposed on families with RPL [1]. Since the pathogenesis is not completely understood, there are few management-related diagnostic and therapy options. Chromosomal and uterine abnormalities, endometrial diseases, endocrine abnormalities, antiphospholipid syndrome, hereditary thrombophilia, immune disorders, genetic causes, environmental determinants, socioeconomic status, and psychosocial stressors have been suggested as causes of RPL [2,3,4].

Therefore, it is emphasized that adequate screening and consistency are necessary for those affected by RPL, in particular, to assess possible dangers and appropriate treatment options [5]. The mutation generated by a change in the position of a single base pair increases prothrombin levels, hence increasing the risk of thromboembolism. Due to MTHFR enzyme inefficiency, two forms of polymorphism have been found. In homozygous people, the efficiency level is significantly lower than average. Consequently, the homocysteine amount rises [6,7].

In other women with placental circulatory diseases, thrombophilia risk factors are more prevalent. In pregnancies associated with slow intrauterine growth, such as in preeclampsia, it is possible to witness late fetal death and abruption placenta [8,9]. Despite contradictory results, many doctors prefer to test women for thrombophilia since there are studies linking thrombophilia with worse pregnancy outcomes. Nevertheless, women with a background of RPL had higher psychosocial and emotional issues during later pregnancies [10,11]; also, couples have had to deal with the accumulated effects of RPL, including increasing fatigue and strain, as well as marital problems caused by consecutive pregnancy miscarriages [12,13].

Consequently, this study anticipated that couples with recurrent pregnancy loss are expected to have an increase in relationship issues, stress levels, and anxiety issues, being unable to fulfil their couple goals of having a family with children. Due to an increased study emphasis, the purpose of this research was to clarify particularly how being diagnosed with thrombophilia impacts women psychologically and how recurrent pregnancy loss correlates with stress levels and marital dissatisfaction.

## 2. Materials and Methods

### 2.1. Study Design and Participants

An observational retrospective cohort study was conducted between January 2021 and January 2022 with patients who enrolled in the study during that time period at the University Clinic of Obstetrics and Gynecology “Bega” affiliated with the “Victor Babes” University of Medicine and Pharmacy from Timisoara. The research population, as well as the pertinent characteristics, were obtained from the clinic’s outpatient population-based administrative database. These records were reviewed by licensed medical professionals who were taking part in the current study.

Bega Clinic, as an auxiliary of Timis County Emergency Clinical Hospital “Pius Brinzeu”, works under the laws of the Local Commission of Ethics that approves Scientific Research and operates in accordance with: (1) Article 167 of Law No. 95/2006, Art. 28, Chapter VIII of Order 904/2006; (2) the EU GCP Directives 2005/28/EC; (3) the International Conference on Harmonisation of Technical Requirements for Registration of Pharmaceuticals for Human Use (ICH); and (4) with the Declaration of Helsinki for recommendations guiding medical practice. The current investigation was given approval on January 20, 2021, identified with the number 27.

### 2.2. Study Inclusion Criteria and Survey Methods

Women with a history of pregnancy were included in the current study based on the definition by the World Health Organization of pregnancy loss, also known as miscarriage [14]. Miscarriage or spontaneous abortion is the most prevalent type of pregnancy loss, being described as the loss of a pregnancy before 20 weeks of gestation by the American College of Obstetricians and Gynecologists (ACOG) [15]. After 20 weeks of gestation, the loss of pregnancy is known as fetal demise [16]. It is estimated that a quarter of all pregnancies and ten percent of clinically diagnosed pregnancies end in miscarriage, while three-quarters of them are known as early pregnancy losses, occurring during the first trimester [17,18]. The first trimester of pregnancy is comprised of the first 14 weeks of pregnancy, respectively, the interval from 14 to 28 weeks represents the second trimester [19].

To examine intimacy issues, stress levels, and relationship satisfaction among women with recurrent pregnancy loss, only female patients with two or more miscarriages were included in the study. For determining the appropriate population size for the research groups, convenience sampling was used. With a margin of error of 5% at a confidence level of 95%, an expected prevalence of thrombophilia in the general population of around 5%, and a pregnancy loss ratio of 10–20% [20], it was projected that at least 381 individuals would satisfy for the sample requirements. It was estimated that the sample would suffice to consist of at least 381 participants.

Thrombophilia was considered as any of the following mutations: factor V Leiden, prothrombin, antithrombin deficiency, protein C deficiency, protein S deficiency, free protein S deficiency, plasminogen activator inhibitor 1 (PAI-1) deficiency, Angiotensin Converting Enzyme (ACE) deletion, Factor VII deficiency, Factor XIII deficiency, β-fibrinogen polymorphism, glycoprotein Ia polymorphism, plasminogen, and tissue-type plasminogen activator deficiency, acquired activated protein C resistance, MTHFR gene mutation. Acquired thrombophilia was not considered for inclusion.

Of the 313 women with thrombophilia and recurrent pregnancy loss who consented to complete the questionnaires, 157 full responses were provided at the deadline of the research interval and were analyzed as the study reference group (cases). The comparison group consisted of female participants with thrombophilia and recurrent pregnancy loss but who had given birth at least once (controls). The control group contained 81 full replies that were evaluated in the current research. Due to incomplete surveys, all incorrect or incomplete information (75 respondents) was eliminated.

The questionnaires were distributed online, and the participants were asked questions on their demographics, history of depression, history of psychiatric medication use, and couple satisfaction. The following five standardized questionnaires translated into the Romanian language were given to the participants: (1) Coping Inventory for Stressful Situations (CISS) [21]; (2) Sexual Interaction Inventory–(SII) [22]; (3) the Dyadic Sexual Communication Scale (DSCS) [23]; (4) the Marital Intimacy Questionnaire (MIQ) [24]; and (5) the Prenatal Psychosocial Profile (PPP) [25].

### 2.3. Variables and Data Sources

An electronic database search and patients’ private records findings contributed to establishing the precise diagnoses of the study participants involved and the status of pregnancy loss in accordance with the International Classification of Diseases (ICD-10) [26]. The variables of interest for the current study comprised: (1) maternal background data–age range, body mass index (BMI), area of residence, relationship status, level of income, level of education, occupation, comorbidities, smoking and alcohol use behavior; (2) obstetrical characteristics–pregnancy attempts, number of pregnancy losses, number of pregnancy-related complications, assisted reproductive techniques, history of STDs, pelvic infections, number of thrombophilia mutations; (3) standardized questionnaires–CISS, SII, DSCS, MIQ, and PPP.

### 2.4. Statistical Analysis

Data analysis was performed using the IBM SPSS software version 27.0 (SPSS. Inc., Chicago, IL, USA). Absolute values and their frequencies were used to represent categorical variables. The proportions were analyzed statistically using the Chi^2^ and Fisher’s exact tests. A Shapiro–Wilk test was performed to assess the normality of data, and the Student’s *t*-test was used to compare means of normally distributed variables. The median and interquartile range was used to describe the non-Gaussian data, which was further compared between groups with the Mann–Whitney U-test. The significance threshold was set for an alpha value of 0.05.

## 3. Results

### 3.1. Patients’ Background

A total of 238 patients were analyzed in the current cross-sectional study. There were 157 completed surveys by women with thrombophilia and recurrent pregnancy loss, respectively 81 completed surveys by women with thrombophilia and recurrent pregnancy loss who achieved a normal pregnancy and preterm or full-term birth. Among these patients, it was observed that relationship status was statistically significantly different, as only 86.6% of patients in the cases group were married, compared with 95.1% in the control group (*p*-value = 0.044). Also, the level of income was significantly higher among patients who achieved pregnancy and gave birth (62.4% vs. 76.5%, *p*-value = 0.027). There was no significant difference in the age, body mass index, and a number of comorbidities of participants. However, it was found that self-reported marital dissatisfaction was significantly higher among patients with thrombophilia with recurrent pregnancy loss compared to those who achieved one birth (15.9% vs. 6.2%, *p*-value = 0.031), as seen in Table 1.

### 3.2. Obstetrical Characteristics

The comparison of obstetrical characteristics between women with thrombophilia and recurrent pregnancy loss (cases), and women with thrombophilia and recurrent pregnancy loss who achieved a normal pregnancy and preterm or full-term birth (controls), are presented in Table 2. It was observed that the reference group had a significantly higher proportion of three or more pregnancy attempts (54.1% vs. 39.5%, *p*-value = 0.032). The proportion of three more pregnancy losses was also significantly higher in the reference group (68.8% vs. 55.6%, *p*-value = 0.043). Associated with the number of pregnancy losses, the number of related complications was also significantly higher among women with thrombophilia who did not give birth. It was also observed that pregnant women with thrombophilia affected by RPL but who successfully gave birth after many attempts accessed significantly more assisted reproductive techniques (64.2% vs. 24.8%, *p*-value < 0.001). Nevertheless, the reference group was affected by significantly more thrombophilia mutations, as the proportion of three or more mutations was 55.4%, compared to only 19.8% in the control group (*p*-value < 0.001).

### 3.3. Analysis of Standardized Questionnaires

**Figure 1:**
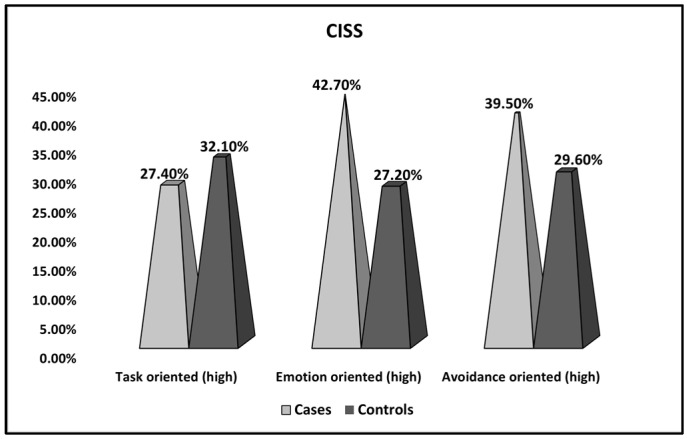
Comparison of CISS survey results between women with thrombophilia and recurrent pregnancy loss (cases), women with thrombophilia and recurrent pregnancy loss who achieved a normal pregnancy, and preterm or full-term birth (controls).

Table 3 and Figure 1 describe the comparison of CISS survey results between women with thrombophilia and recurrent pregnancy loss (cases) and women with thrombophilia and recurrent pregnancy loss who achieved a normal pregnancy and preterm or full-term birth (controls). The standardized questionnaires showed multiple significant differences between the two study groups. While examining the CISS survey results presented in Table 3 and Figure 1, it was observed that patients in the reference group were more likely to be emotion-oriented (42.7% vs. 27.2%, *p*-value = 0.019).

**Figure 2:**
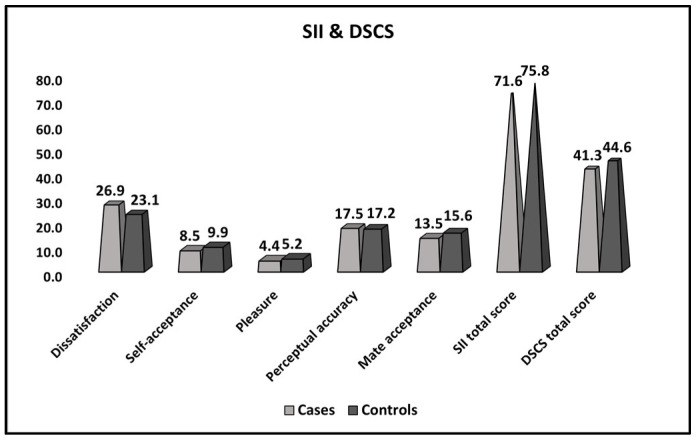
Comparison of SII and DSCS survey results between women with thrombophilia and recurrent pregnancy loss (cases), women with thrombophilia and recurrent pregnancy loss who achieved a normal pregnancy, and preterm or full-term birth (controls).

Table 4 and Figure 2 present the comparison of SII and DSCS survey results between women with thrombophilia and recurrent pregnancy loss (cases–reference group), women with thrombophilia and recurrent pregnancy loss who achieved a normal pregnancy, and preterm or full-term birth (controls). Among the significant differences, it was observed that women in the reference group had higher levels of dissatisfaction, and lower levels of self-acceptance, pleasure, and marital quality scores, while the perceptual accuracy was similar to the control group. The total sexual interaction instrument score (SII) was statistically significantly lower in the reference group (71.6 vs. 75.8, *p*-value = 0.003). Similar to the SII score, the dyadic sexual communication total score (DSCS) was significantly lower among those who failed to give birth (41.3 vs. 44.6, *p*-value = 0.002).

**Figure 3:**
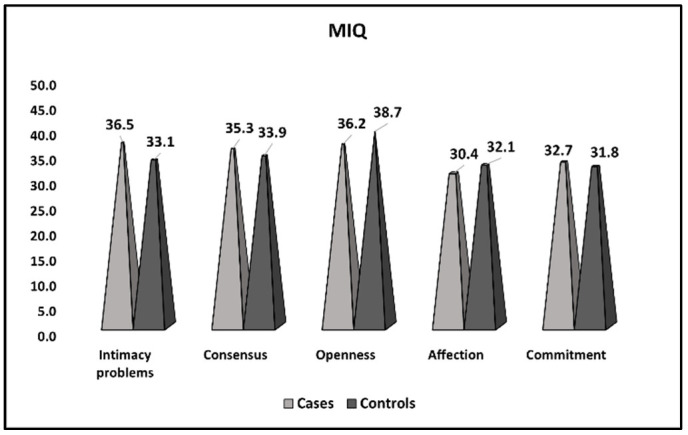
Comparison of MIQ survey results between women with thrombophilia and recurrent pregnancy loss (cases), women with thrombophilia and recurrent pregnancy loss who achieved a normal pregnancy, and preterm or full-term birth (controls).

Table 5 and Figure 3 describe the comparison of MIQ survey results between women with thrombophilia and recurrent pregnancy loss (cases) and women with thrombophilia and recurrent pregnancy loss who achieved a normal pregnancy and preterm or full-term birth (controls). It was observed that women from the reference group had significantly bigger intimacy problems and lower openness scores than women in the control group (36.5 vs. 33.1, *p*-value = 0.008), respectively (36.2 vs. 38.7, *p*-value = 0.019). Consensus levels, affection, and commitment were similar in both study groups.

**Figure 4:**
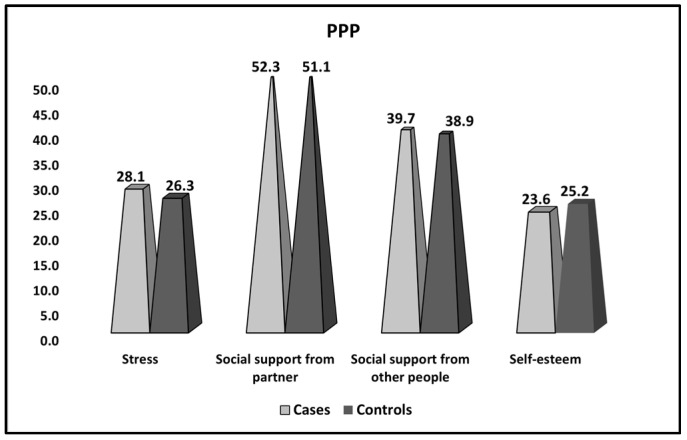
PPP survey results between women with thrombophilia and recurrent pregnancy loss (cases), women with thrombophilia and recurrent pregnancy loss who achieved a normal pregnancy, and preterm or full-term birth (controls).

The prenatal psychosocial profile was analyzed between women with thrombophilia and recurrent pregnancy loss (cases), women with thrombophilia and recurrent pregnancy loss who achieved a normal pregnancy and preterm or full-term birth (controls), and presented in Table 6 and Figure 4. It was observed that social support from partners and from other people was equivalent in both groups, although stress levels were significantly higher in the reference group (28.1 vs. 26.3, *p*-value = 0.004), respectively, self-esteem levels were significantly lower (23.6 vs. 25.2, *p*-value = 0.020).

## 4. Discussion

### 4.1. Literature Analysis

This study revealed significant data about the correlation between women’s views, perceptions of marriage satisfaction, intimacy issues, and stress levels with the diagnosis of thrombophilia with recurrent pregnancy loss. Among the important results from these respondents was the observation that they participated in sexual behavior less often. Additionally, it was found that the women in the reference group who did not give birth had a significantly higher proportion of three or more pregnancy attempts (54.1% vs. 39.5%) and a significantly higher proportion of three more pregnancy losses (68.8% vs. 55.6%). It was observed that patients in the reference group were more likely to be emotion-oriented (42.7% vs. 27.2%). Also, women in the reference group had higher levels of dissatisfaction and lower levels of self-acceptance, pleasure, and marital quality scores. The total SII and DSCS scores were significantly lower than women with thrombophilia with a history of RPL who eventually gave birth. Women from the reference group had significantly greater intimacy problems and stress levels while having lower openness scores and self-esteem scores than women in the control group.

RPL not only creates a great deal of psychological and financial struggles for the concerned women and their spouses, but it also affects the couples’ relationships and the way they interact with their family and relatives [13,27]. Failed marriages are more likely for women with RPL than for those without losses or with live births [28]. There is no known explanation, and research has been restricted. According to studies, women have a larger desire to discuss their losses than their male partners, and these discrepancies may lead to unhappiness and strain on the relationship [29]. In addition, males often take a significant role, supporting their relationships and exhibiting resilience in times of difficulty. This reduces their capacity to demonstrate emotional sensitivity when it is required of them and may cause them to hide their own emotions and needs [30]. There is also evidence that couples, after a miscarriage, may have sexual difficulties. In contrast, the relationship may also be a protective factor for mental health, as spousal engagement and marital fulfillment are connected with reduced anxiety and depression scores after a single pregnancy loss or RPL [31].

Regarding active social support, observers often misunderstand the impact of RPL and, in particular, the challenges they constitute for males [32]. According to research, 41% of the women surveyed were dissatisfied with the responses of their friends and family following miscarriages and accused of a lack of empathy and attention [29]. This is concerning since social care from family and friends is recognized as a protective factor for mental health and is related to reduced levels of depression and anxiety in women with RPL. Little is known about the degree of family and friend assistance that women with recurrent pregnancy loss and their partners seek to receive [33]. Two qualitative studies from the United States and Australia provide preliminary evidence that the support of friends and family members with comparable circumstances is seen as beneficial by women who have had one or more miscarriages and their spouses [34]. Offers to cook meals or care for youngsters from friends and families, as well as offers of time off and assistance from businesses and churches were highly appreciated. Proposals to anticipate future pregnancies and efforts to emphasize good elements of the miscarriages were seen as callous, as were remarks suggesting that the women’s lives and/or choices may have contributed to their losses.

Another research study showed that women with RPL and severe depression had worse sexual function scores, which is consistent to our findings where patients with RPL had significantly more intimacy problems [35,36]. Due to the fact that emotional and sexual difficulties are not identified by health care providers because fertility concerns are prioritized or because such difficulties are considered taboo, the evaluation of sexual and mental health must be included in the consultation for women with a history of RPL, regardless of whether the patient pursues help for depression and sexual dysfunction.

Although thrombophilia and the severity of mutations are known to significantly impact the occurrence of miscarriage, it is also hypothesized that several socio-economic factors can directly or indirectly affect this risk. Studies confirm that unhealthy lifestyle habits, lower levels of education, and number of habitual abortions are more frequent among women from lower socio-economic classes [37,38,39]. Therefore, the socioeconomic status of a woman with thrombophilia might further increase the risk of recurrent miscarriage.

Regarding the influence of thrombophilia on the success of pregnancy, in our study, it was observed that the reference group was affected by significantly more thrombophilia mutations, as the proportion of three or more mutations was 55.4%, compared to only 19.8% in the control group. It was also observed in our study that pregnant women with thrombophilia affected by RPL but who successfully gave birth after many attempts accessed significantly more often assisted reproductive techniques (ART) (64.2% vs. 24.8%). Other studies reported that ART is associated with a higher risk of thrombo-embolic events, therefore increasing the risk among patients with thrombophilia [40].

A recent comprehensive analysis indicated that the antepartum risk of VTE after IVF is twice that of the typical pregnant population as a whole. VTE was always related to pregnancy, while arterial thrombosis was documented earlier and even in the absence of pregnancy. Less than forty percent of patients had concomitant thrombophilia. This higher risk of VTE in IVF pregnancies is a result of a five- to tenfold greater risk in the first trimester [41]. However, the existing evidence does not support a correlation between thrombophilia and ART results [42]. Consequently, the ease of access to ART among these individuals might readily fix their issues. Considering the possibility of a link between congenital thrombophilia and unfavorable pregnancy outcomes, anticoagulation has only been demonstrated to be advantageous in the context of antiphospholipid syndrome (APS) [43].

### 4.2. Strengths and Limitations

A first limitation of the study is that the sample size requirements were not entirely met, as the computed optimal size of the sample was 381 participants, while only 238 patients were ultimately included. Therefore, it may be essential to collect additional examples to obtain appropriate statistical power. Also, control samples were not matched; therefore, confounding variables could not be controlled for. Thus, a matched sample may provide more precise findings. As a second limitation, the cross-sectional design may be regarded as an important constraint since it does not give a convincing evaluation and assessment in time of the participants’ stress levels and relationship difficulties. Since the surveys only examine the participants’ responses at a single moment in time, it is impossible to identify with precision the levels of stress and pair satisfaction. As a third limitation, the use of questionnaires may result in a high subjectivity index from all respondents who agreed to complete them, which might lead to many biases in the acquired data. In conclusion, the results of the current study can only be extrapolated to the community that was studied due to the likelihood that religion and culture, as well as other population-specific characteristics, might have an influence on the observations.

## 5. Conclusions

It is likely that marital dissatisfaction is significantly higher among women with thrombophilia with recurrent pregnancy loss compared to other women with thrombophilia and recurrent pregnancy loss but who eventually achieved one birth. Since the financial status of those who achieved pregnancy, and the rate of assisted reproductive techniques was higher in the latter group, it is advisable to target families affected by infertility due to thrombophilia and other causes in order to facilitate their access to ART interventions. By doing this, the affected families will achieve their goals, which will likely reduce the dissatisfaction levels, divorce rates, and stress levels in these families.

## Figures and Tables

**Table 1:** Comparison of background characteristics between women with thrombophilia and recurrent pregnancy loss (cases), women with thrombophilia and recurrent pregnancy loss who achieved a normal pregnancy, and preterm or full-term birth (controls).

Variables	Cases (*n* = 157)	Controls (*n* = 81)	*p*-Value
Age (≥35 years)	71 (45.2%)	29 (35.8%)	0.162
BMI (>25kg/m^2^) **	34 (21.7%)	12 (14.8%)	0.205
Area of residence (urban)	112 (71.3%)	59 (72.8%)	0.807
Relationship status (married)	136 (86.6%)	77 (95.1%)	0.044
Level of income (average or higher)	98 (62.4%)	62 (76.5%)	0.027
Level of education (higher education)	92 (58.6%)	54 (66.7%)	0.225
Occupation (employed)	117 (74.5%)	58 (71.6%)	0.628
Substance use behavior			
Frequent alcohol consumption	7 (4.5%)	5 (6.2%)	0.566
Frequent smoker	22 (14.0%)	13 (16.0%)	0.674
Chronic comorbidities			0.819
None	107 (68.2%)	52 (64.2%)	0.715
1	37 (23.6%)	23 (28.4%)	
≥2	13 (8.3%)	6 (7.4%)	
Others			
History of depression since pregnancy problems occurred	11 (7.0%)	4 (4.9%)	0.533
Use of psychiatric medication since pregnancy problems occurred	18 (11.5%)	9 (11.1%)	0.934
Self-reported marital dissatisfaction	25 (15.9%)	5 (6.2%)	0.031

Data reported as n (frequency) and calculated using the Chi-square test and Fisher’s exact unless specified differently; ** Weight measured when pregnancy loss occurred; BMI–Body mass index; Chronic comorbidities include cardiovascular disease, metabolic disease, autoimmune disease, respiratory disease, and chronic infections.

**Table 2:** Comparison of obstetrical characteristics between women with thrombophilia and recurrent pregnancy loss (cases), women with thrombophilia and recurrent pregnancy loss who achieved a normal pregnancy, and preterm or full-term birth (controls).

Variables	Cases (*n* = 157)	Controls (*n* = 81)	*p*-Value *
Pregnancy attempts			0.032
2	72 (45.9%)	49 (60.5%)	
≥3	85 (54.1%)	32 (39.5%)	
Number of pregnancy losses			0.043
2	49 (31.2%)	36 (44.4%)	
≥3	108 (68.8%)	45 (55.6%)	
Number of previous pregnancy-related complications			<0.001
None	9 (5.7%)	23 (28.4%)	
1	111 (70.7%)	35 (43.2%)	
≥2	37 (23.6%)	23 (28.4%)	
Assisted reproductive techniques	39 (24.8%)	52 (64.2%)	<0.001
History of STDs	18 (11.5%)	6 (7.4%)	0.324
Pelvic infections	25 (15.9%)	14 (17.3%)	0.788
Number of thrombophilia mutations			<0.001
1	26 (15.3%)	7 (44.4%)	
2	46 (29.3%)	10 (35.8%)	
≥3	87 (55.4%)	37 (19.8%)	

* Chi-square or Fisher’s exact test; STD–Sexually transmitted disease; Pregnancy-related complications include anemia, preeclampsia, maternal infections, deep venous thrombosis, and pulmonary embolism.

**Table 3:** Comparison of CISS survey results between women with thrombophilia and recurrent pregnancy loss (cases), women with thrombophilia and recurrent pregnancy loss who achieved a normal pregnancy, and preterm or full-term birth (controls).

Items (Score Range)	Cases (*n* = 157)	Controls (*n* = 81)	*p*-Value
Task-oriented (high)	43 (27.4%)	26 (32.1%)	0.447
Emotion-oriented (high)	67 (42.7%)	22 (27.2%)	0.019
Avoidance-oriented (high)	62 (39.5%)	32 (29.6%)	0.133

Data reported as n (frequency) and calculated using the Chi-square test and Fisher’s exact unless specified differently. CISS–Coping Inventory for Stressful Situations. CISS is a questionnaire that examines various coping strategies for stressful circumstances. Using a Likert scale, its statements target several aspects of human conduct as either negative or positive affirmations. The range is from 1 (never) to 6 (always). The CISS provides three scores: task-oriented, emotion-oriented, and avoidance-oriented. Task-oriented refers to a person’s concentration on finishing a task. Each subscale consists of sixteen distinct components. The higher the score on the scale, the greater the intensity of the coping technique.

**Table 4:** Comparison of SII and DSCS survey results between women with thrombophilia and recurrent pregnancy loss (cases), women with thrombophilia and recurrent pregnancy loss who achieved a normal pregnancy, and preterm or full-term birth (controls).

Items	Cases (*n* = 157)	Controls (*n* = 81)	*p*-Value
Dissatisfaction	26.9 ± 2.7	23.1 ± 2.8	<0.001
Self-acceptance	8.5 ± 1.4	9.9 ± 1.6	<0.001
Pleasure	4.4 ± 2.3	5.2 ± 2.0	0.008
Perceptual accuracy	17.5 ± 4.3	17.2 ± 5.5	0.644
Mate acceptance (marital quality)	13.5 ± 2.0	15.6 ± 2.2	<0.001
SII total score	71.6 ± 10.5	75.8 ± 9.6	0.003
DSCS total score	41.3 ± 8.0	44.6 ± 7.9	0.002

Data reported as mean ± SD and calculated using Student’s *t*-test; SII–Sexual Interaction Instrument; DSCS–Dyadic Sexual Communication Scale.

**Table 5:** Comparison of MIQ survey results between women with thrombophilia and recurrent pregnancy loss (cases), women with thrombophilia and recurrent pregnancy loss who achieved a normal pregnancy, and preterm or full-term birth (controls).

Items	Cases (*n* = 157)	Controls (*n* = 81)	*p*-Value
Intimacy problems	36.5 ± 9.8	33.1 ± 8.4	0.008
Consensus	35.3± 8.1	33.9 ± 7.9	0.203
Openness	36.2 ± 7.6	38.7 ± 8.1	0.019
Affection	30.4 ± 6.6	32.1 ± 6.7	0.063
Commitment	32.7 ± 6.9	31.8 ± 6.7	0.336

Data reported as mean ± SD and calculated using Student’s *t*-test; MIQ–Marital Intimacy Questionnaire.

**Table 6:** PPP survey results between women with thrombophilia and recurrent pregnancy loss (cases), women with thrombophilia and recurrent pregnancy loss who achieved a normal pregnancy, and preterm or full-term birth (controls).

Subscales	Cases (*n* = 157)	Controls (*n* = 81)	*p*-Value
Stress	28.1 ± 4.2	26.3 ± 5.0	0.004
Social support from partner	52.3 ± 10.5	51.1 ± 9.8	0.393
Social support from other people	39.7 ± 7.4	38.9 ± 8.1	0.445
Self-esteem	23.6 ± 4.9	25.2 ± 5.2	0.020

Data reported as n (frequency) and calculated using the Chi-square test and Fisher’s exact unless specified differently. PPP—Prenatal Psychosocial Profile.

## Data Availability

The data presented in this study are available on request from the corresponding author.
